# Central serous chorioretinopathy and angioid streaks: coincidental?

**DOI:** 10.1186/s12886-022-02566-w

**Published:** 2022-09-05

**Authors:** Susana Costa Penas, José António Resende, Amândio Rocha Sousa, Ângela Veloso Carneiro, Fernando Falcão Reis

**Affiliations:** 1 Ophthalmology Department, Centro Hospitalar Universitário de São João, E.P.E., Alameda Professor Hernâni Monteiro, 4200-319 Porto, Portugal; 2 Faculty of Medicine of University of Porto, Porto, Portugal; 3 Ophthalmology Department, Clínica Médica Arrifana de Sousa, Penafiel, Portugal

**Keywords:** Central serous chorioretinopathy, CSC, Angioid streaks, OCTA

## Abstract

**Background:**

To report an unusual case of central serous chorioretinopathy in a patient with angioid streaks.

**Case presentation:**

The authors describe a case report of a 26-year old male patient presenting acute scotoma and metamorphopsia in OD. He had been diagnosed with angioid streaks complicated with choroidal neovascularization and referred to us for treatment. The patient presented an ETDRS score of 85 letters (20/20) in OD and in OS. The anterior segment examination was unremarkable. Fundoscopy revealed bilateral angioid streaks (AS) and peau d’orange, as well as a small neurosensory retinal detachment in the macula of OD. A multimodal retinal analysis, including fundus photography, infra-red and fundus autofluorescence imaging, spectral-domain optical coherence tomography, optical coherence tomography angiography, fluorescein and indocyanine green angiography was performed. The diagnosis of central serous chorioretinopathy was made in the absence of any identifiable choroidal neovascularization. He was submitted to half-dose photodynamic therapy with verteporfin. One month later, he reported no visual complaints, his vision was 85 letters (20/20) in OD and a complete resolution of the sub-retinal fluid was registered. No signs of choroidal neovascularization were detected on the optical coherence tomography angiography (OCTA). A complete medical workup evaluation was made to exclude systemic diseases usually associated with AS.

**Conclusions:**

To the authors’ knowledge, this is the second reported case of CSC associated with angioid streaks. The focal abnormalities in the Bruch’s membrane and the irregular vascular choriocapillary network associated with AS might predispose to CSC.

## Background

Central serous chorioretinopathy (CSC) is a disorder characterized by the occurrence of one or multiple serous retinal detachments, resulting from the increased hydrostatic pressure caused by a thickened and hyperpermeable underlying choroid. It has been included in the pachychoroid spectrum of diseases, featuring areas of compressed choriocapillaris and Sattler's layers by dilated Haller vessels (pachyvessels) [1].

Angioid streaks (AS) are crack-like dehiscences of an abnormal Bruchs membrane (BM), described as calcified and brittle, presenting as dark reddish brown irregular linear bands, typically radiating from the optic disc toward the periphery [2]. It is considered a hereditary retinal disease, due to a reported familial tendency, although the inheritance pattern still remains unknown. It is frequently associated with some systemic diseases as Pseudoxanthoma Elasticum, Paget disease, Ehlers–Danlos syndrome, Marfan syndrome, hemoglobinopathies, and hypercalcemia. Its onset is rare on the first decade, usually manifesting between the second and fifth decades [2, 3]. A significant impairment in visual acuity may occur in case of secondary choroidal neovascularization (CNV) or sub-retinal hemorrhage or due to the growth of the AS towards the fovea [3].

## Case presentation

A 26-year-old healthy male patient was referred to us presenting acute scotoma and metamorphopsia in OD. He had been recently diagnosed with bilateral angioid streaks and presumable choroidal neovascularization and submitted to intravitreal treatment with aflibercept with no response.

**Fig. 1:**
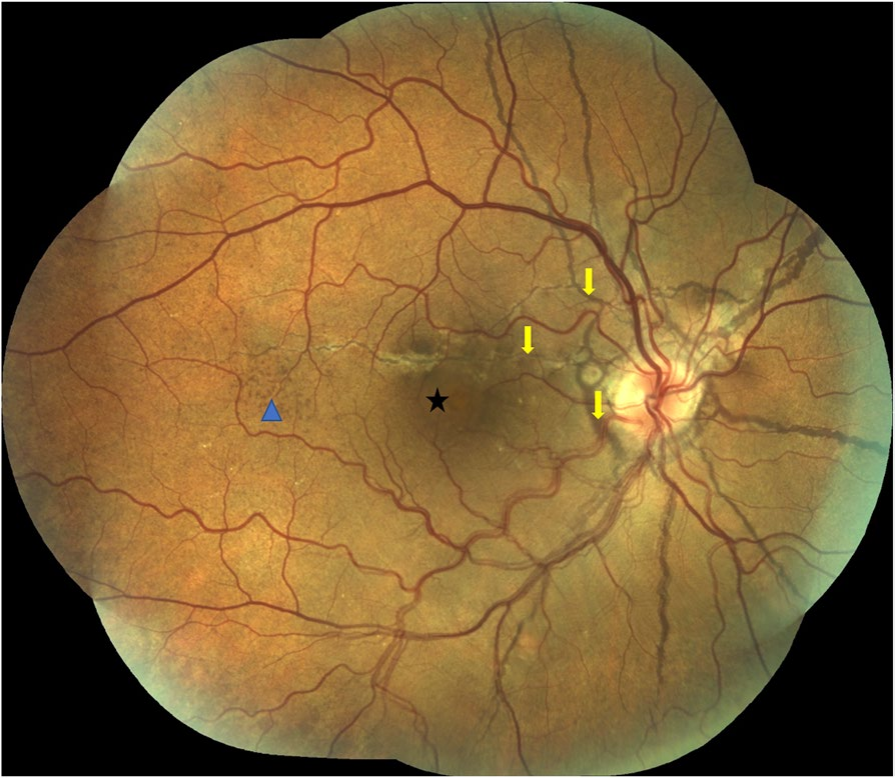
Colour fundus photograph montage of the right eye. Multiple brownish angiod streaks are visible radiating from the optic disc (yellow arrows). A small serous detachment is visible in the central macula (black star). Peau d’orange is present in the temporal mid-periphery (blue arrowhead)

**Fig. 2:**
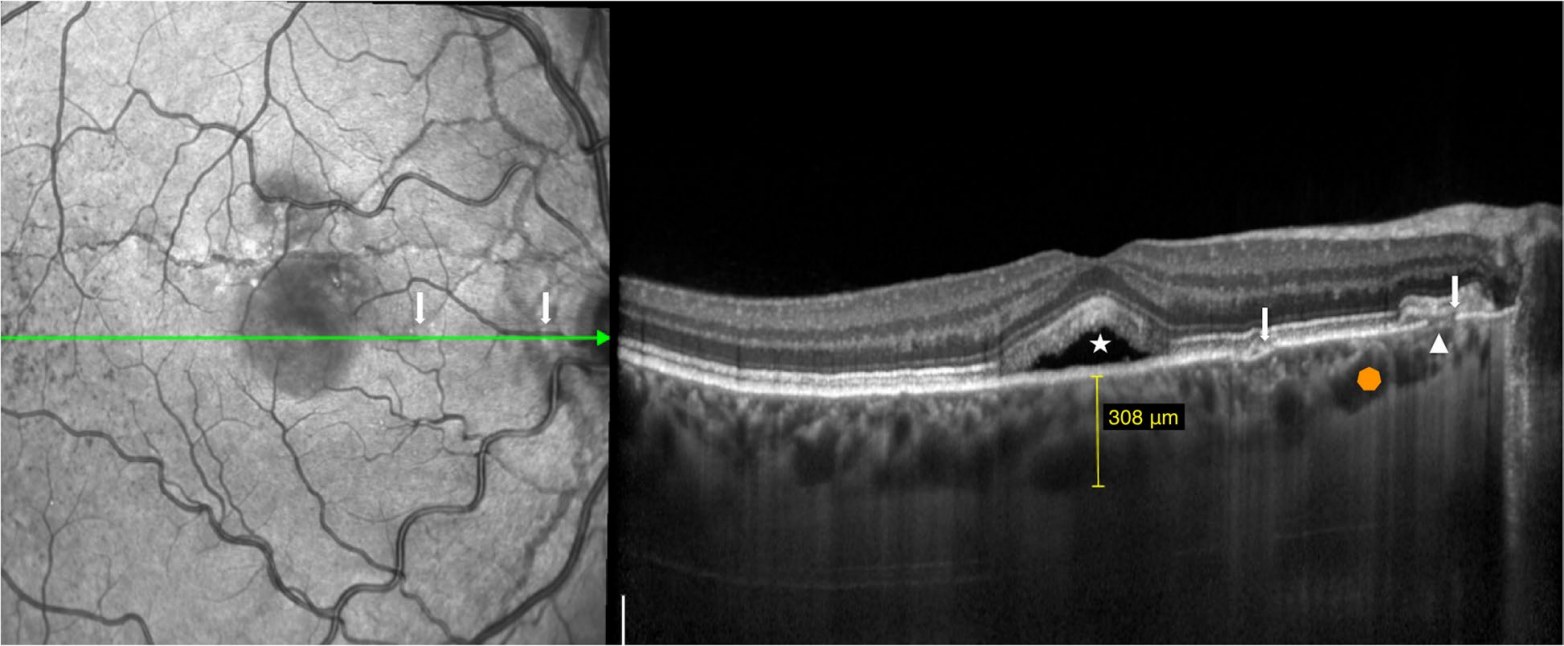
An horizontal optical coherence tomography scan crossing the fovea shows a small neurosensory retinal detachment (white star), with some shedding of the photorreceptors’ outer segments. An interruption of the retinal pigment epithelium and Bruch’s membrane is visible in correspondance with the angioid streaks (arrowhead). Some hyperreflective material is seen above the RPE (white arrow), probably corresponding to fibrovascular tissue scaring. Although the choroid is not particularly thick (sub-foveal thickness of 308 μm), it presents some dilated Haller vessels with attenuation of the inner choroid (orange octagon), suggesting a pachychoroid feature

**Fig. 3:**
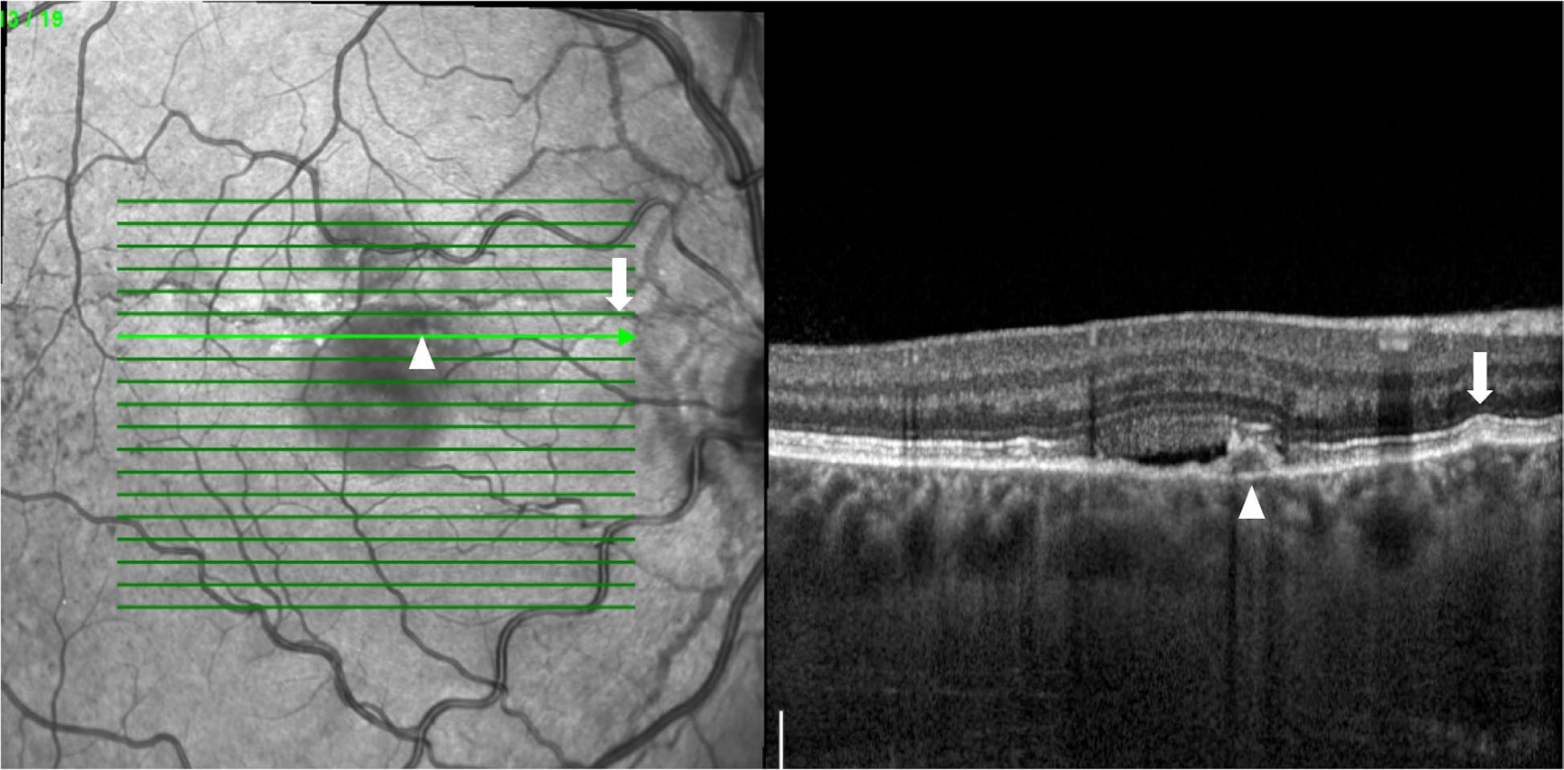
Optical coherence tomography showing a small retinal pigment epithelium (RPE) detachment underlying the sub-retinal fluid, corresponding to the leaking spot (arrowhead). A small deflection of the Bruch’s membrane and RPE (arrow) results from a small angioid streak, visible in the infra-red image on the left

**Fig. 4:**
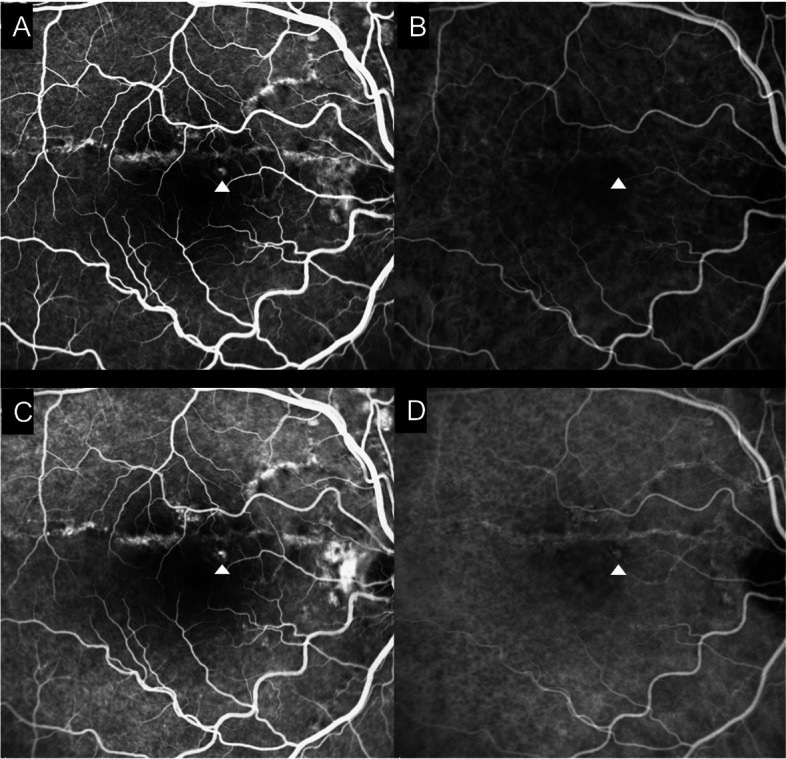
A multimodal angiographic evaluation enhances a small hyperfluorescent spot (white arrowhead) on the early phase fluorescein angiography (FA) (**A**), not contiguous with the angioid streak (AS), and not visible in the indocyanine green angiography (ICG) (**B**). Late phase angiograms show mild FA leakage (**C**), with no hot-spots on the ICG (**D**). The AS are hyperfluorescent, but more visible in the FA than in the ICG

**Fig. 5:**
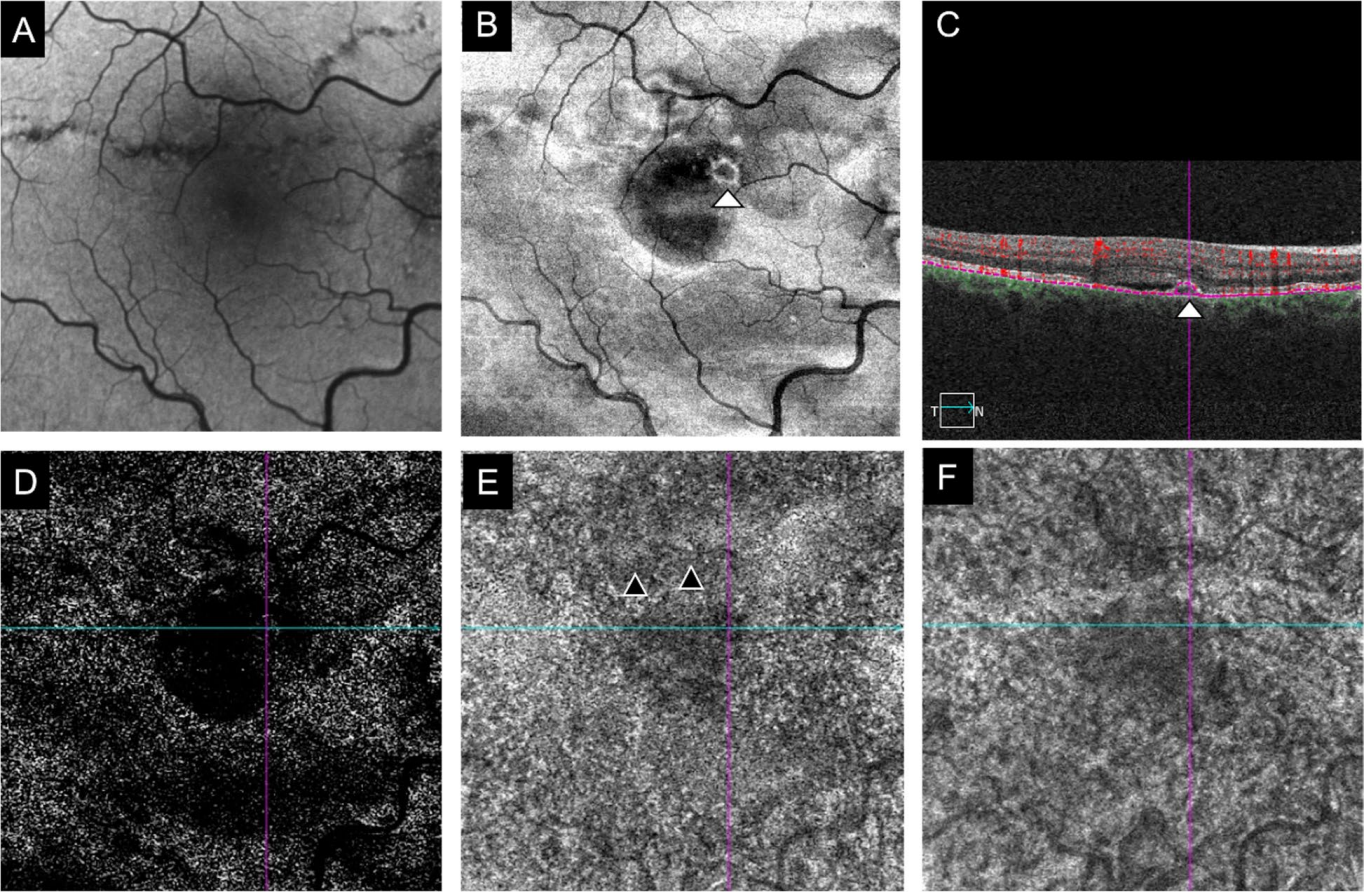
Optical coherence tomography angiography (OCTA) imaging. The infra-red image (**A**) and the structural enface OCT (**B**) highlight the angioid streaks (AS) and sub-retinal fluid location. The leaking spot (white arrowhead) is visible both in enface (**B**) and cross-sectional OCT (**C**), showing no signs of intralesional vascular flow (**C**). OCTA images at the avascular (**D**), choriocapilaris (**E**) and choroidal (**F**) layers show no signs of neovessels. A slight choriocapilaris rarefaction is seen in the AS vicinity (black arrowhead)

**Fig. 6:**
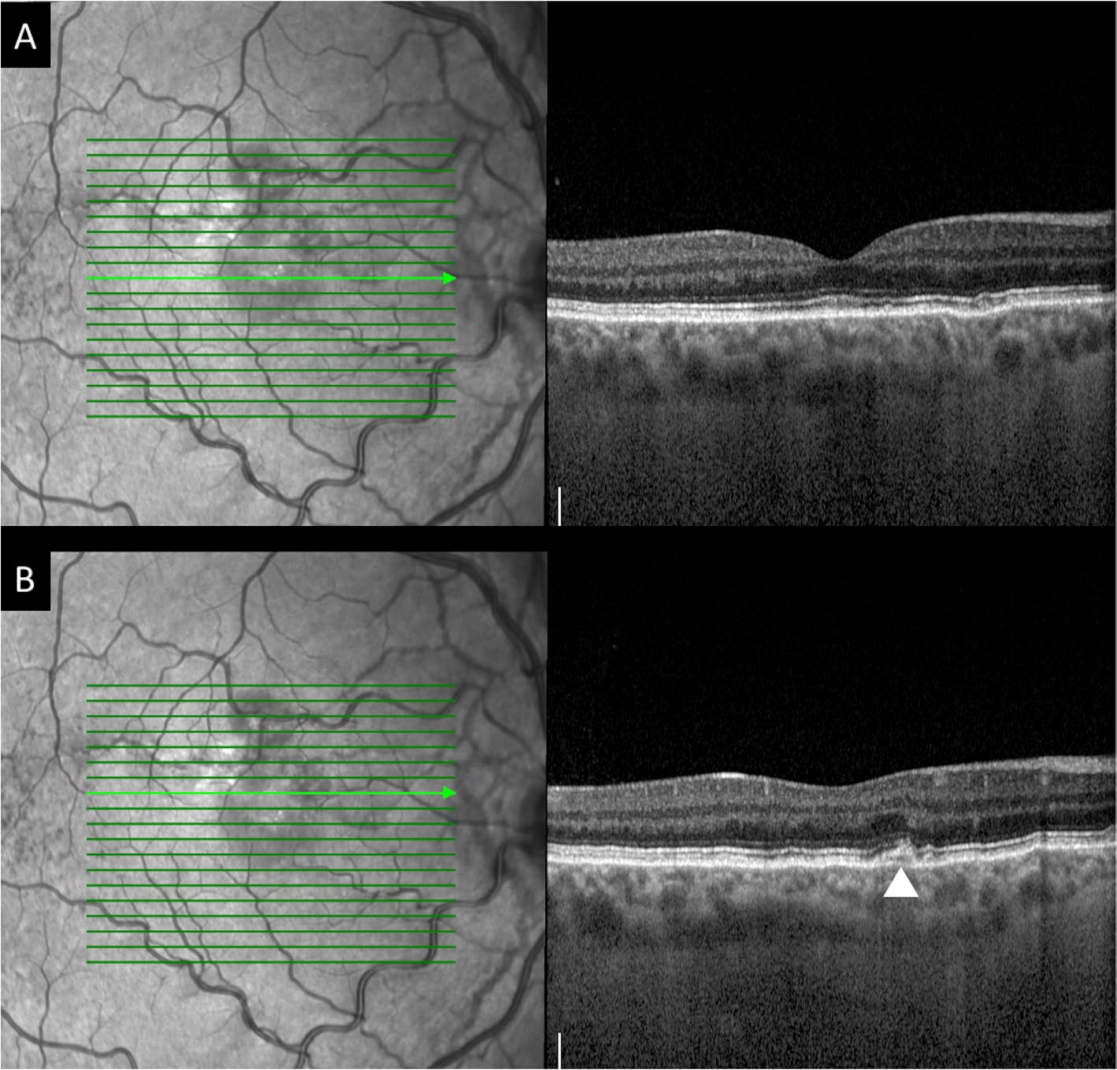
After half-dose photodynamic therapy, the serous retinal detachment resolved completely on the optical coherence tomography foveal-centered scan (**A**) and there where no signs of sub or intra-retinal fluid around the previous leaking spot (arrowhead) (**B**)

At presentation his best-corrected visual acuity (BCVA) using the ETDRS score was 85 letters (20/20) in OD and in OS. The anterior segment examination was unremarkable and his intraocular pressure was 12 mmHg in both eyes. Dilated fundoscopy revealed bilateral angioid streaks (AS) and peau d’orange, as well as a small serous neurosensory retinal detachment in the macula of OD (Fig. 1). A multimodal retinal analysis including fundus photography, infra-red and fundus autofluorescence imaging, spectral-domain optical coherence tomography (SD-OCT), fluorescein (FA) and indocyanine green angiography (ICGA) was performed using the Spectralis system (Spectralis +HRA; Heidelberg Engineering, Heidelberg, Germany). An optical coherence tomography angiography (OCTA) (AngioPlex CIRRUS HD-OCT Model 5000, Carl ZeissMeditec, Inc., Dublin, OH) was also used with a scanning area of 3 mmx3 mm, centered on the macula. SD-OCT was performed with and without the enhanced-depth imaging technique (EDI), presenting a small serous macular detachment overlying a small retinal pigment epithelium (RPE) detachment in OD, near to an angioid streak, with no signs of chronicity in the outer retina or underlying RPE (Figs. 2 and 3). No sub or intra-retinal fluid was detected in OS. The subfoveal choroidal thickness was measured using the software manual caliper, presenting 308 μm in OD and 411 μm in OS. Pachyvessels were identified bilaterally, compressing the choriocapillaris (Fig. 2). The FA revealed an active leaking spot near but not contiguous to an angioid streak, with no leaking in the ICGA (Fig. 4). No signs of choroidal neovascular complex were detected on the OCTA, presenting no flow underneath the small RPE detachment on the structural OCT (Fig. 5) but some minor vascular network irregularities were detected at the choriocapillaris layer in both eyes (Fig. 5). The diagnosis of acute central serous chorioretinopathy was made, and he was submitted to half-dose photodynamic therapy (HD-PDT) with verteporfin. One month later, he reported no visual complaints, his vision ETDRS score was 85 (20/20) in OD and a complete resolution of the sub-retinal fluid was registered (Fig. 6). No signs of choroidal neovascularization were detected on the OCTA.

A complete medical workup evaluation was made to exclude systemic diseases usually associated with the AS, including the evaluation by a dermatologist, cardiologist and geneticist. No systemic disease was detected so far, but the genetic study result is still pending. The family history was negative for angioid streaks.

## Discussion/ conclusions

To our knowledge, this is the second reported case of CSC associated with angioid streaks, and although both pathologies have been widely studied, their co-presentation seems unusual [4].

After a careful analysis of the patients' records and images, we made the diagnosis of CSC and excluded a small AS-related CNV considering the lack of response to previous anti-VEGF therapy, absence of leakage on the ICGA, absence of a type 2 CNV lesion on the OCT, presence of pachychoroid features on SD OCT, absence of an identifiable CNV lesion and absence of blood flow inside the lesion on OCTA. Furthermore, although both CSC and AS CNV respond to PDT, it is not plausible that an hypothetical neovascular lesion, that did not respond to anti-VEGF, would completely resolve with just one half-dose PDT treatment, showing no recurrence during follow-up.

Therefore, considering a potential relation between CSC and angioid streaks, we must focus on their pathophysiology. Recent studies using OCTA highlighted the choriocapillaris rarefaction on OCTA in patients with AS without CNV, interpreting this finding as an eventual atrophy of this vascular layer, associated with the overlying crack-like breaks in a thickened BM [5–7]. In particular cases, they described an irregular vascular network in the choriocapillaris in areas affected with AS, presenting a flat elevation of the RPE and hyperreflective accumulations between the BM and RPE on simultaneous structural SD-OCT [5]. They speculated that this irregular vascular network might result from the development of a fibrovascular tissue over the crack-like breaks in the calcified BM, as a natural attempt to repair the damaged BM and overlying RPE [5].

It is nowadays known that BM works as a natural barrier between the choroidal circulation and the outer retina, limiting the flow of fluid, particles and growth factors across these two structures, therefore, its integrity is vital to keep both compartments physiologically sealed. Recent OCTA studies have revealed an increased choroidal vascular flow in both affected and unaffected eyes in CSC patients [8]. In patients with an already damaged BM and RPE, as in the presence of AS, the physical rupture of this barrier, unable to yield an increased hydrostatic choroidal pressure, seems rather plausible and expected. In fact, an RPE break is a common feature in both CSC and AS, and is co-localized with FA hyperfluorescence, either by leakage in an active lesion or by a window defect in an atrophic one [9]. Furthermore, several cases of polypoidal choroidal vasculopathy (PCV), another pachychoroid spectrum disease, have been reported as secondary to AS [10, 11]. Baillif-Gostoli et al [11] even hypothesize that the common histopathologic lesions encountered in AS secondary to pseudoxanthoma elasticum and PCV suggests that their co-existence might not be coincidental. Moreover, PCV associated to chronic CSC is not a rare finding [12].

We therefore hypothesize that the focal abnormalities in the Bruch’s membrane and the irregular vascular choriocapillary network associated with AS might predispose to CSC, and the co-manifestation of these diseases might not be entirely coincidental. The lack of more reported cases might be due to an underestimation of CSC occurrence in these patients, whose complaints of blurred vision and metamorphopsia might be attributed to the angioid streak itself or misdiagnosed as secondary CNV. The more microstructural data we get from the developing imaging technologies, the more knowledge we get on the pathophysioloy of retinal diseases.

In conclusion, a careful multimodal imaging assessment in AS patients is crucial to achieve a correct diagnosis and timely treatment.

## Data Availability

All data generated or analyzed during this study are included in this published article [and its supplementary information files].

## Abbreviations

AS: Angioid streaks
BCVA: Best-corrected visual acuity
BM: Bruchs membrane
CNV: Choroidal neovascularization
CSC: Central serous chorioretinopathy
EDI: Enhanced-depth imaging technique
FA: Fluorescein angiography
HD- PDT: Half-dose photodynamic therapy
ICGA: Indocyanine green angiography (ICGA)
OCTA: Optical coherence tomography angiography
PCV: Polypoidal choroidal vasculopathy
RPE: Retinal pigment epithelium
SD-OCT: Spectral-domain optical coherence tomography
